# Diabetes

**Published:** 1867-09

**Authors:** H. Bence Jones

**Affiliations:** Late Physician to St. George’s Hospital


### DIABETES.

By H. BENCE JONES, A.M., M.D., F.R.S., late Phy'n to St. George's Hospital.

(*Lectures on Pathology and Therapeutics.*)

Dr. Jones, in speaking of the treatment of diabetes, in his elaborate work on Pathology and Therapeutics, says, that the effect of diet is far beyond that of any known remedy. An anti-farinaceous, or in other words, an anti-saccharine diet, will remove the sugar from the urine, and stop all the symptoms of the complaint in all those cases in which the power of consuming the animal sugar remains unaffected. Even when the consumption of the animal sugar is imperfect or impossible, an anti-saccharine diet will lessen the thirst, the flow of water, the dryness of the mouth, and even the constipation, and check, though it may not stop, the waste. The simplest formula for the diet may be thus stated. All animal produce, including fish, flesh, fowl, game, eggs, cream, and meat-soup, should be taken; and all vegetable food that contains starch, dextrine, and sugar should be avoided. The vegetable substances that contain most starch, dextrin, and sugar are rice, maize, arrowroot, sago, potatoes, oatmeal, peas, beans, biscuit, toast, maccaroni, vermicelli, and all confectionery. Fruits are even worse than vegetables. Apricots, plums, peaches, cherries, pears, and gooseberries are nearly as bad, and some worse, than rice or maize. Stout, porter, and ale, cider, port, madeira, champagne, and sherry are more or less highly saccharine; cocoa and chocolate contain nearly 20 per cent. of starch and dextrine naturally, and more is often added.

As regards the use of medicines in diabetes, Dr. Jones is of opinion that there are two ends to be gained by their use; the first and most important is to promote the oxidation of the sugar; or, failing this, to compensate the system for the loss of saccharine fuel, and the consequent loss of power and nutrition by promoting the supply and oxidation of the oleaginous fuel. Of all the medicines that can be given for the promotion of the oxidation, whether of sugar or fat in the body, iron and alkalies are the most energetic; and hence, beyond all other remedies, iron or the ammonia-citrate of iron with excess of ammonia, or with other alkalies, are usually the best medicines for diabetes. The iron may be given in potass or Vichy, or in Fachingen water, and that preparation which confines the bowels least is most to be preferred. Hence, the potassio-tartrate and Griffith's mixture are often useful. Alkalies without iron promote oxidation. Soda or potass may be given in the caustic state, or as carbonates. Carbonate of ammonia in ten, fifteen, or twenty grain doses thrice daily, in any gaseous mineral water, lessens the thirst.

Besides alkalies some animal substances are thought to promote change in the sugar in diabetes. Of these rennet and pepsine may be mentioned; but Dr. Jones is not satisfied that either are very useful.

Vegetable and animal oils and fats constitute important remedies in diabetes. Of all these cod-liver oil and cream are most frequently used. The following case may be taken as an instance of the amount of cod-liver oil that can be given:—

A man, aged twenty-four years, was admitted into St. George's Hospital, having lost two stone in weight during eight months. He passed seven quarts of urine daily. He remained under treatment for a month, during which time he was on animal diet and cod-liver oil. He began with half an ounce daily, and this was gradually increased up to eight ounces. The quantity of urine fell to two pints and a half, specific gravity 1030, and he increased in weight from 8st. 8℔. to 9st. 1℔.

Cream may be given in any quantity until the tongue begins to be coated, then it soon disagrees, and the stomach refuses to take it, or rejects it when taken.

Pure glycerine may be employed as a substitute for sugar in tea, and in other liquids.

To lessen the thirst, and the craving for food, opium is very useful—five or ten grains of Dover's powder, or five or ten drops of laudanum, may be given once or twice daily.

The second great object in the treatment of diabetes is to remove the constipation.

Notwithstanding the amount of food eaten, the action of the bowels usually is very difficult. All saline aperients increase the thirst, and pass off by the urine. Magnesia, from the absence of acidity, is usually inactive. Castor oil is by far the best aperient, when it does not nauseate, then capsules containing castor oil, with minute quantities of croton oil, are most efficacious. Compound extract of colocynth with jalapine, scammony, or gamboge, or podophyllin, will act when oil cannot be taken. Calomel may be used as an aperient, but it has not any advantage over other chemical or mechanical irritants to the mucous membrane of the bowels.

